# A novel approach to identify critical knowledge gaps for food safety in circular food systems

**DOI:** 10.1038/s41538-024-00265-y

**Published:** 2024-06-19

**Authors:** Stefan P. J. van Leeuwen, A. M. Verschoor, H. J. van der Fels-Klerx, M. G. M. van de Schans, B. J. A. Berendsen

**Affiliations:** Wageningen Food Safety Research (WFSR), Wageningen University & Research, Akkermaalsbos 2, 6708 WB Wageningen, The Netherlands

**Keywords:** Risk factors, Agriculture

## Abstract

The transition from linear production towards a circular agro-food system is an important step towards increasing Europe’s sustainability. This requires re-designing the food production systems, which inevitably comes with challenges as regards controlling the safety of our food, animals and the ecosystem. Where in current food production systems many food safety hazards are understood and well-managed, it is anticipated that with the transition towards circular food production systems, known hazards may re-emerge and new hazards will appear or accumulate, leading to new -and less understood- food safety risks. In this perspective paper, we present a simple, yet effective approach, to identify knowledge gaps with regard to food safety in the transition to a circular food system. An approach with five questions is proposed, derived from current food safety management approaches like HACCP. Applying this to two cases shows that risk assessment and management should emphasize more on the exposure to unexpected (with regards to its nature and its origin) hazards, as hazards might circulate and accumulate in the food production system. Five knowledge gaps became apparent: there’s a need for (1) risk assessment and management to focus more on unknown hazards and mixtures of hazards, (2) more data on the occurrence of hazards in by-products, (3) better understanding the fate of hazards in the circular food production system, (4) the development of models to adequately perform risk assessments for a broad range of hazards and (5) new ways of valorization of co-products in which a safe-by-design approach should be adopted.

## Introduction

Current European food production is not sustainable in the long term. Fossil and mineral resources are depleted. Changing climate is leading to increased drought eventson the one hand and more heavy rainfall on the other hand. Also the ever-increasing world population leads to an increased food demand. The transition towards a circular agro-food system, rather than current non-circular production supply chains, is an inevitable step towards increasing sustainability, respecting the planetary boundaries. At all stages of food production, distribution and consumption, stakeholders such as scientists, policy makers, farmers, food producers, consumers are re-thinking and re-designing the current food production approaches. The European Commission has set ambitious goals under the European Green Deal of which the New Circular Economy Action Plan (CEAP)^1^ and the Farm to Fork strategy^2^ are the main building blocks. They include the reduction of the use of artificial fertilizers, reduction of nutrient and food losses, growth of organic farming, water reuse, etc. At every level (farm level, food supply chain level, consumer level) and at all scales (local, regional, global) changes are currently taking place and many more future changes are expected. The transition towards a circular food system is a systemic change, and it is expected that the production of food in 10–20 years from now will be significantly different from current approaches.

Recently, Muscat et al.^3^ proposed five ecological principles (safeguard, avoid, prioritize, recycle and entropy) to guide biomass use towards a circular bioeconomy. Considering application of these principles, severe changes in the European food production system are to be expected and - according to recent reviews^4,5^—these may go along with the occurrence of potential new food safety risks. Three of these principles are likely to impact food safety. Examples are given here.

*Safeguard* - This principle prevents the depletion of natural sources and thus bans the use of artificial fertilizers. In combination with an expected decreasing number of livestock in Europe, and thus availability of manure, this results in a demand for alternative products to provide the required nutrients for agricultural practice. These nutrients are vastly available in products we currently consider as being waste, such as sewage sludge. Thus, sewage sludge is an interesting candidate for future use for agricultural application (most likely after treatment). The reintroduction of such a product may be accompanied with the introduction of a whole range of legacy and new chemical hazards, e.g., human pharmaceuticals, personal care products and contaminants like heavy metals, plant protection products and persistent organic pollutants (POPs), including per- and polyfluoroalkyl substances (PFASs).

*Prioritize* - This principle states that products should be used as high in the food chain as possible. Thus, if a product is suited for human food, it should be used as such and not as animal feed. As a result, lower quality products will be available for, for instance, animal feed. The use of lower quality products at higher levels in the food chain may pose new food safety hazards. Furthermore, new processing techniques to upcycle co-products, here defined as by-products from production processes, might also induce new food safety hazards.

*Recycle* - This principle focusses on the reuse of products in food production that are currently being considered as waste. A clear result is that more co-products and waste streams are recycled and introduced in e.g., animal feed or compost. The reintroduction of such products can yield new food safety hazards. An example is the increased use of intra- or inter-species of animal co-products. The use of processed animal proteins from mammals in feed of cattle could transmit prions causing bovine spongiform encephalopathy (BSE). Also, low concentrations of contaminants might be reintroduced as these co-products are only to a minor extent systemically monitored.

Evidently, new food safety challenges arise in the transition to more circular food production. Legislation is not in all cases fit-for-purpose for a circular food production system. In some cases regulation hampers the transition, in other cases the regulations are not fully designed to prevent new food safety issues from occurring. Regulation 2020/741^6^ was implemented recently, but only focuses on measures for safe application from a microbiological hazards point of view, but not for chemical hazards. The main food safety risks seem to originate from the (re)introduction of low(er) quality products or products that are currently considered waste into the food system^4,5^. In this perspective paper, we present a simplified risk assessment and management approach, based on HACCP principles, consisting of five questions. We choose to start from the HACCP approach since HACCP is already mostly applied in an operational setting by food and feed stakeholders (from production to distribution). We have adopted the HACCP approach for assessing food safety in a circular food production system and with aiming to identify knowledge gaps therein. In addition to feed/food producers, it can be applied by scientists/technologists designing new circular processes, and by risk assessors/managers who wish to determine potential risks of new development in circular food & feed production. The effectiveness of this novel approach is demonstrated based on two currently relevant case studies related to circular food production to identify critical knowledge gaps, highlight points of concern and assess current barriers.

### Approach

#### Five question approach

**Fig. 1:**
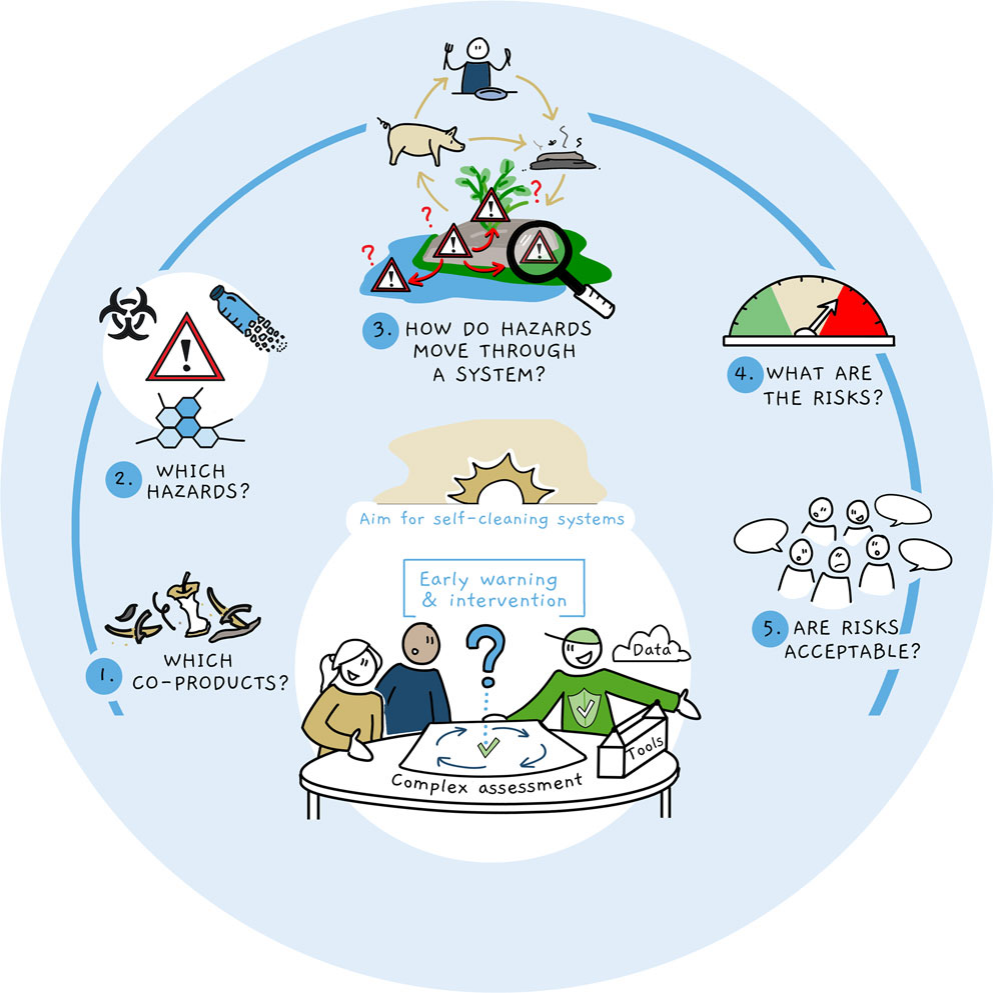
Five-step approach for safety assessment in a circular food production system. 1: Input—determine which (waste) material or co-product is used in the circular food production system. 2: Hazards—identify the hazards that occur in the circular food production system. 3: Fate—determine how do these hazards move through the circular food production system. 4: Risks—determine the risks of these hazards in the circular food production system. 5: Acceptability—determine if the risk is acceptable or if risk management measures need to be taken. Copyright Bureau voor Beeldzaken, 2022.

To asses food safety in the transition to a circular food production system, we here propose an effective approach that allows detection of critical knowledge gaps. This approach is based on five aspects that need to be addressed; elements touched upon by these five questions are also covered by the EU legislation of the General Food Law^7^ and HACCP approaches. In traditional risk assessment (that come in various approaches^8,9^, the main steps are (1) hazard identification, (2) hazard characterization, (3) exposure assessment and (4) risk characterization. These steps were the basis of the presented approach, yet additional attention is given to the exposure assessment. More specifically, this relates to the input of by-products in the circular system and the behavior of the hazards in the circular food-production system. The five questions are shown in Fig. 1.

Q1: Input deals with defining the case and can involve the following aspects: What raw materials and co-products are incorporated in the production and distribution process? Where do they come from? Here, the scale of the process should also be considered. Does the process consider a single farm, a region or the whole country or planet? Small scale processes can lead to very amplified risks for a specific region. Large scale processes could potentially harm a large group of consumers.

Q2: Hazard deals with the identification and definition of the (un)expected hazards: What potential hazards can be present in these raw materials and co-products? Is legislation available? Can and will an input control monitoring strategy be applied? Monitoring data are required to sufficiently answer this question.

Hazards can be divided into three types: chemical, biological and physical. Chemical hazards may include chemical compounds, such as (heavy) metals, plant protection products, pharmaceuticals (for animals and humans), natural toxins (mycotoxins, plant toxins, marine toxins), environmental contaminants (dioxins, polychlorinated biphenyls (PCBs), PFASs) and process contaminants (e.g., mineral oil saturated hydrocarbons (MOSH) and mineral oil aromatic hydrocarbons (MOAH). Biological hazards include all organisms, like bacteria, and protozoa, as well as biologically active particles such as prions and viruses, that can be harmful to human health. Physical hazards are for instance (micro)plastics, metal particles and bone fragments, as well as radioactive particles.

Hazards can originate from two different sources: (1) hazards that are intentionally applied in food production (so called ‘residues’) and hazards that (2) unintentionally (and sometimes unknowingly) are introduced into the food system (‘contaminants’). The intentionally applied hazards include pharmaceuticals and plant protection products. Hazards that are unintendedly introduced usually originate from co-products of any sort or environmental contamination.

**Fig. 2:**
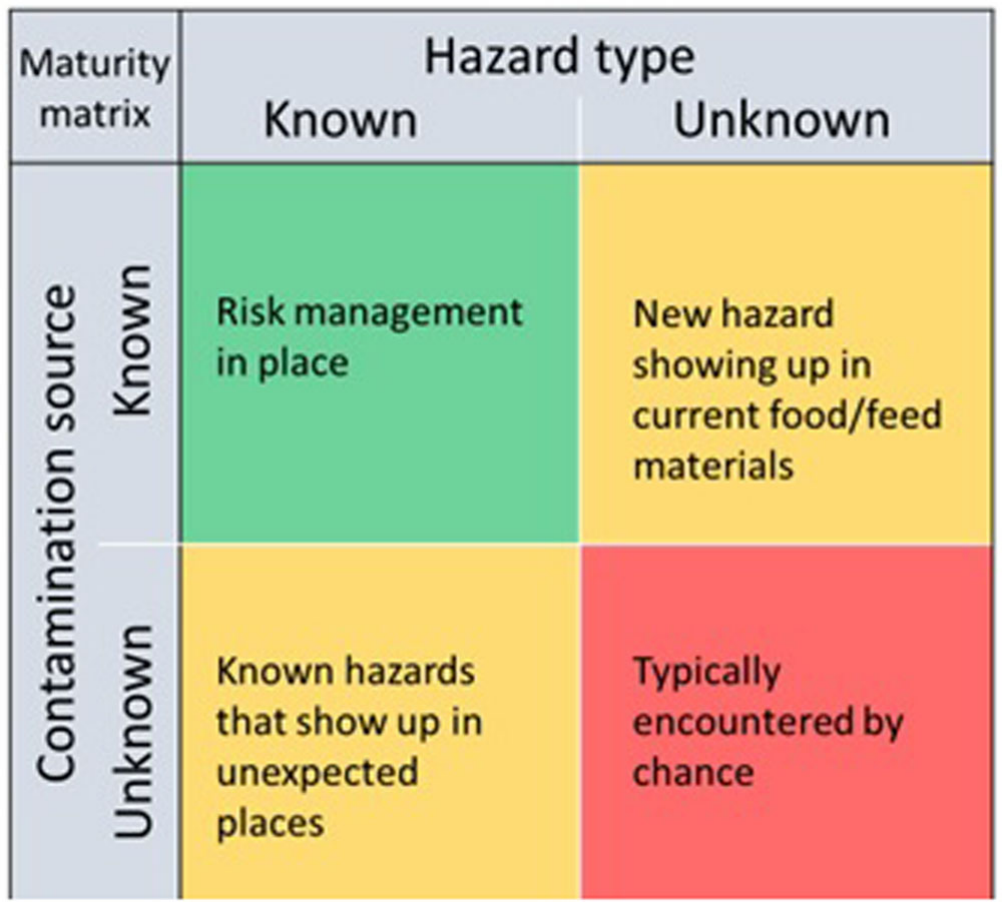
The knowns and unknowns: where hazards can show up expectedly and unexpectedly. Top-left: known hazards show up in known food/feed production situations; top-right: in known food/feed production situations previously unknown hazards show up; bottom-left: in previously unknown food/feed production situations known hazards show up; bottom-right: in previously unknown food/feed production situations previously unknown hazards show up. See text for explanation and examples.

The different types of hazards may have a different nature, and the level of knowledge and regulation differs substantially. For example, ‘known’ hazards may appear in unexpected places, whereas in other cases ‘unknown’ hazards may show up in known cases. Figure 2 shows a categorization of knowns and unknowns as regards the nature and the origin of the hazard. A further explanation is given below.

*Known hazards* from *known sources* are numerous. These include, but are not limited to, marine toxins in shellfish, veterinary medicines in animal co-products and plant protection products in animal feed. For *known hazards* from *known sources*, typically appropriate risk management practices are in place. Legal maximum limits, such as laid down for example in Commission Regulation 2023/915^10^ (succession of regulation EC/1881/2006), other limits and control programs are in place and are continuously reviewed based on the latest scientific insights.

A case of a *known hazard* arising from an *unknown source* as a result of recycling was the case of medroxyprogesterone acetate (MPA) scandal in the early 2000s. A glycose containing co-product originating from the manufacturing of birth-control medication was used in pig feed. In a large number of pigs infertility was diagnosed. Only then it was found that the animal feed contained high concentrations of MPA, a substance that is banned in Europe for animal treatment. Thousands of animals were destructed^11^. For this case, one could advocate that the co-product was per definition a high-risk product that had been introduced into the food system.

An example of an *unknown hazard* originating from a *known source* is well illustrated by the case of mineral oil saturated hydrocarbons (MOSH) and mineral oil aromatic hydrocarbons (MOAH) in recycled paper and carton board used as food contact material. Although it is known that (recycled) food contact materials may contain certain contaminants^12^, the presence of MOSH/MOAH in recycled paper and carton board was long neglected. This issue was discovered already in the early 2010s^13,14^, but it was propelled only after a non-governmental organization (NGO) brought this to greater public attention. This resulted in several measures at the food producer side and authorities to adequately identify and manage risks, ultimately leading to reduction of the dietary exposure to these contaminants.

The most intriguing category is the category with *unknown hazards* in a yet *unknown situation*. An example of such a case evolved from 2006 onwards in Germany, where the application of per- and polyfluoralkyl substances (PFAS) polluted soil conditioners led to a widespread PFAS contamination in the catchment areas of the rivers Moehne and Upper-Ruhr. By that time, there was only little known about the hazards of PFASs (*unknown hazard*), and the occurrence in soil conditioners (*unknown situation*), which resulted in an uncontrolled pollution event. Because of the uncertainty, drastic measures were implemented to reduce undesirable human exposure of citizens in the impacted area. Later, a similar event occurred in the south of Germany (Rastatt area)^15–17^.

Q3: Fate deals with the translocation and persistence of the food safety hazards. For the translocation it involves the following aspects: Do the hazards stay in the compartment (e.g., animal, soil, crop, water) they are introduced into or do they move among compartments? If so, to which compartments? It is important to understand how hazards move among compartments from a mechanistic point of view. The fate of a hazard depends on specific circumstances, including the properties of the contaminant and the environment the contaminant is in. For instance, the transmission of contaminants from soil to water depends on the soil composition, but also on the type of contaminant itselve^18^.

Regarding the persistence: are the hazards degraded to a no-effect concentration by the system (natural mitigation) or are they persistent, or transformed to other bioactive hazards? In an ideal situation, a hazard is mitigated by the system itself, e.g., a substance is degraded/deactivated in soil. The persistence of a hazard should be known as it is an important parameter in understanding the fate. Persistent hazards, e.g., DDT or microplastics, remain in the system and might accumulate. If these hazards currently show no negative effects, they may do so in time, whether that is in five or 100 years from now. Data need to be obtained and should be made publicly available to allow a meaningful risk assessment. Note that degradation of, for instance, a chemical hazard can result in the transformation of the hazard, still (or even more) exerting a negative effect. An example is estradiol, a female hormone, that can be transformed in soil to the transformation product estrone, a more estrogenic substance^19^. Wastewater treatment may also result in other transformation products that show (eco)toxicological potential^20–22^. Note that some hazards can exert an acute risk (e.g., allergens in food products) and as such their degree of persistence is subordinate.

Q4: Risk assessment deals with the quantification or estimation of the risk and involves questions such as: Do the hazards yield a potential risk (one health perspective) in the compartments they can occur in? Risk studies cannot be carried out for all hazards, in all compartments for all possible toxicological endpoints. Therefore, it is important to understand which hazards can be present in which compartment. As such, risk studies should focus on specific hazards in specific compartments for specific endpoints. To complicate risk assessment, especially in a circular food production system, combinations of hazards can occur, which need specific attention.

Q5: Risk management deals with all the aspects of risk management, and involves questions like: Are such potential risks permissible (from a policy and consumer perspective) and can such potential risks be actively mitigated, preferably at the source? Here also the potential interaction among hazards needs to be considered, e.g., how the exposure to chemical contaminants can result in a higher impact of a microbial infection.

### Application and knowledge gaps

To get a better understanding of the complexity of the topic and to identify knowledge gaps using the presented approach, the approach is applied to two different cases with a focus on unknown and known biological and chemical hazards.

### Case 1: Former foodstuffs to animal feed

Instead of going to waste, former foodstuffs are increasingly being utilized in animal feed. In the EU, annually 5 million tonnes of former foodstuffs are already being used for feed^23^. Until now, products of primarily plant origin are being valorized at a large scale into animal feed. The foodstuffs that are currently being used are products that in principle are/were also suitable for human consumption, but may not be sold as such (anymore), such as cutting remains, leftovers from product development, products over expiry date, or other products that do not meet the quality standards established. Collection and conversion of former foodstuffs into feed is in Europe coordinated by a few large processing companies, having dedicated suppliers, that process these products into feed products. These processes are now restricted to relatively safe former foodstuffs originating from bakeries and confectionery factories but from a sustainability point of view, as well as in order to address the growing need for proteins, reuse of foodstuffs of animal origin, such as milk, eggs, and meat that for some reason are not suitable (anymore) for human consumption (e.g., because of commercial reasons, quality, production failures etc.), should be considered in addition to the current plant-based materials.

Strict EU legislation for the use of animal proteins as animal feed is in place since the start of the 21st century in order to gain control over the spread of BSE as well as the spread of certain contagious animal diseases in Europe. These include three types of restrictions being: (a) the ruminant ban (protein of ruminants is not allowed for use as feed), (b) the extended feed ban (animal proteins may not be used in several other applications), and (c) the species to species ban (anti-cannibalism; proteins from one species may not be fed to the same species). Though some exemptions are in place, these regulations to a large extent hamper the use of animal derived proteins as animal feed. About twenty years later, the European Commission is considering some a relaxation of the extended feed ban to enable a more circular production system. Since August 2021, processed pig proteins are allowed to be used as chicken feed, and processed chicken proteins are allowed for use in pig feed, and eight insect species are currently allowed for inclusion in animal feed (EC 2021/1372)^24^.

A further relaxation could be to allow using so-called swill (kitchen refuse/waste) in feed. These waste streams are generated in every household, but for logistical reasons, only (larger) kitchens and caterers are considered realistic sources. The approach with the five questions will help to identify the threats and opportunities.

Q1: Input: Former foodstuffs, particularly swill (kitchen refuse/waste) for animal feed. Primary source for these waste streams are (larger) restaurants and caterers. Collection and processing will typically be organized at a local or regional scale.

Q2: Hazard: The primary hazards associated with swill are biologically active particles (prions; BSE/TSE) and pathogens/zoonoses (e.g., foot and mouth disease, Aujeszky’s disease, African swine flu)^25,26^. Swill not only has the risk of transmitting animal diseases, but also diseases that eventually can affect human beings, such as Creutzfeld-Jacob disease, a rare spongiform encephalitis (brain disease), suspected to be caused by consumption of BSE-infected beef. For these reasons, current EU legislation does not allow inclusion of swill in animal feed^27^. Also for that reason monitoring programs for forbidden anima proteins in animal feed are in place.

Q3: Fate: The reuse of swill bears the risk of introducing novel hazards into the animal production chain^26,28^. Such hazards may circulate and/or accumulate in the food system. Viruses and prions may be re-introduced via swill and may spread among animals and transfer from animals to humans. Particularly prions are notoriously resistant to digestive enzymes, heat, disinfectants and desiccation and strongly bind to solids such as feed or soil particles. Bound to particles, prions can stay infectious for many years and may as such remain present as a hazard in the system. Lack of proper removal mechanisms may even lead to accumulation in the food chain.

Q4: Risk: Due to the variety of sources and processing options, a true risk assessment can only be performed on a case-by-case basis. It is obvious that animal proteins within swill currently bear unacceptable risks. A complicating factor here is that in many current kitchen and catering practices, all swill (both plant and animal based) is disposed as mixed waste. This means that the mixture carries a larger risk than some of its individual constituents would. For example, fruit and vegetable waste, which commonly is the largest part of swill, bears zero-risk for transmitting animal-related biological hazards. However, from a precautionary principle, once in a mixture with more hazardous animal waste, the whole mixture should be treated as high risk material. Would plant-based swill be collected separately, this would be easier to reuse in animal feed and human foods.

Q5: Risk management: The current ban on swill application in animal feed has been a very effective measure to reduce possible health risks to a minimum. However, in view of the current ambition to reduce food waste and close production loops, other options to utilize swill should be explored. This calls for adequate risk governance approaches.

Knowledge gaps. This case relates to a *known hazard in a known situ*ation (Q2, Fig. 2). The application of our proposed approach reveals that current legislation is effective in reducing risks to an absolute minimum. For animal proteins, legislation has proven to be effective: between 2005 and 2015 about 73,000,000 cattle were tested for BSE in the EU, and in this period the number of reported cases dropped from 554 cases in 2005 to just two in 2015^29^.

However, current EU legislation hampers the further utilization of former foodstuffs such as swill on a larger scale. From a food security point of view, alternative options should be investigated.

Relaxation of the established feed bans should therefore be accompanied by case-based risk assessments performed on extensive scientific basis, as has been done prior to the most recent partial lift of the extended feed ban^30^. Such an approach should enable to make informed decisions on acceptable risks, although this can only be done for known hazards and known sources.

We conclude that, for the case of the reintroduction of swill into the food production chain, knowledge gaps are mainly related to risk management (Q5). Reducing potential risks of swill starts with better separation at the source. A promising start could be to explore the options of relatively clean streams within the catering waste.

As evident from the difficulties involved with mixed waste streams mentioned before, separation of swill into fractions with higher (e.g., meat, bones) or lower risks (e.g., fruit & vegetables, fries, bread) could be an option to manage these risks more precisely. It is not clear yet to what extent such a separation can be achieved in practice. The practical issues regarding separation and collection should be further explored.

Another step would be the investigation of the most promising routes towards valorization, such as to allow its use for certain dedicated purposes such as feeding it to particular animals (including insects), conversion into microbial proteins (fermentation) or breakdown into valuable components (biorefinery).

### Case 2: Water reuse as a water source in irrigation in agriculture

Wastewater products comprise a suite of materials originating from urban and industrial wastewater treatment plants (WWTP). Urban and industrial wastewater is, next to being a waste stream, also a valuable source of fresh water and nutrients. When mentioning wastewater here, we also consider gray water and excreta^31^. The European Commission (EC) aims at increasing the reuse of treated wastewater from a current 3% to 15% in a couple of years^32^ in order to battle freshwater shortages. Wastewater mining deals with the reuse of wastewater products, e.g., reclaimed water, sludge, struvite and other materials which are valuable products thereof. Agriculture is an important (future) recipient of wastewater products for the purpose of irrigation and fertilization. Countries and regions like the state of California, Spain and Israel are lacking sufficient resources of freshwater already and face drought issues frequently or even (semi)continuously. They have turned to reclaimed water for irrigation of lawns and public gardens (California) or agricultural production fields (e.g., Spain, Israel). Wastewater is known to be vulnerable to contain various pathogens and chemical hazards, and these may comprise risks when agricultural reuse is considered. The safe reuse of reclaimed water for agricultural irrigation from a microbiological hazards point of view was recently regulated^6,33^. Here, we limit the assessment to the chemical hazards of wastewater reuse. Considering its nature, a plethora of chemical compounds occur in wastewater^8,34,35^, sometimes referred to as contaminants of emerging concern (CEC). These include pharmaceutical products (e.g., pain relievers, blood pressure medications, anticonvulsant medication, antibiotics), personal care products (e.g., detergents like quaternary ammonium compounds and musk fragrances like galaxolide), coloring agents from textiles (e.g., indigo dyes), industrial products (e.g., PFASs), plant protection products and household chemicals. These contaminants, when present in reclaimed water, may present potential hazards when applied in agriculture for irrigation. In this respect, it may compromise the safety of food products. Some studies have demonstrated the uptake and deposition of contaminants from reclaimed water into crops, such as residues of pharmaceuticals in vegetables^36–38^ and PFAS in plants^39,40^. One may perceive that the occurrence of such contaminants would a priori rule out the use of reclaimed water. But given the intrinsic value in terms of water and nutrients, it is crucial to investigate if, and how reclaimed water can be applied in agriculture providing safe foods.Recently published legislation and guidelines^6,33^ provides criteria for application of reclaimed water. However, these criteria focus primarily microbiological hazards, although it is acknowledged that large knowledge gaps exist on the risk of CECs in agricultural produce.

The five question approach is posed for the case of water reuse using reclaimed water for irrigation purposes to identify critical knowledge gaps. However, as the number and variety of hazards in this case is very large, this cannot be done in brief. Furthermore, for many hazards insufficient data are currently available for an adequate assessment. To demonstrate the applicability of the presented approach, we limited the scope of this case to the potential presence of PFAS in wastewater. Though, note that this only covers a small part of the potential risks.

Q1: Input: The aim is to increase the use of reclaimed water from domestic origin for irrigation on a global scale. Currently reclaimed water is applied for irrigation in specific countries^41^, but not yet on a global scale.

Q2: Hazard: Many different microbiological, pathogenic or chemical hazards can be present in (treated) wastewater. Some are known and others are unexpected and unknown. Even though the unexpected hazards need consideration, here we focus on a known one. Therefore, the focus is on chemical hazards only, and specifically on PFASs. This group of chemicals contains over thousands of substances of which, according to current knowledge, a limited number, including perfluorooctanoic acid (PFOA) and perfluorooctanoic sulfonate (PFOS), are considered the most relevant.

Q3: Fate: Some studies investigated the fate of PFASs in WWTP water^42,43^. Current wastewater treatment is insufficiently effective for complete removal of PFASs. Consequently, PFASs remain in the reclaimedwater. When applying this water on agricultural land, the PFASs will be in direct contact with the crops and/or be mixed with the topsoil. Depending on the PFASs characteristics and soil composition, the PFASs can (1) adsorb to soil, immobilizing the PFAS^44^, (2) leach to surface and/or ground water^45,46^, (3) run-off or leach in a rainfall event or (4) be taken up by crops^39,40^. Currently the extent to which they might be taken up by crops is not well understood but this process clearly depends on the physico-chemical characteristics of the PFASs, on the soil characteristics and on the crop species and/or variety^39,40^. When taken up by crops, the PFASs may end up in animal feed or human food. Also, PFASs that leach to surface water might, depending on their physical-chemical properties, accumulate in fish^47^, through which humans can be exposed. Currently, data and models to predict the fate of the individual PFASs after land application are not available. Especially the perfluoroalkyl carboxylic acids and perfluoroalkyl sulfonic acids (e.g., PFOS and PFOA) are known to be very persistent. They will not degrade under natural conditions and as such accumulate in soils and foods. Furthermore, a recent study showed that PFASs precursors, e.g., 8:2 fluorotelomer sulfonic acid, can partly be transformed to legacy PFASs like PFOA during wastewater treatment adding to the PFASs burden.

Q4: Risk: PFASs are deemed to exert a negative health effect at extremely low concentrations. The current state of knowledge is that several PFASs have, among other health effects, a negative effect on the immune system^48^, and current human dietary exposures in the EU lead to unacceptable risks^48^. This implies that the exposure to PFAS could result in an increased susceptibility for microbial infections in animals and humans. Effects of PFASs on soil and aquatic life ecosystems are vastly unknown.

Q5: Risk management: With the current knowledge, developing risk management, including elements like risk mitigation and risk monitoring is sensible to do. Mitigation can be of technological or regulatory nature.

Knowledge gaps. This case is related to a mostly *known hazard in an unknown situation* (Q2, Fig. 2), when specifically scoped to PFASs. However, in a broader perspective, it relates to *unknown hazards in an unknown situation* (Fig. 2) as the nature of all relevant PFASs when reusing wastewater in food production is currently not well understood.

As regards the current regulatory tools, guidelines on minimum requirements for water reuse were recently adopted^6,33^, setting uniform minimum water quality requirements for the safe reuse of reclaimed water in agricultural irrigation. However, these guidelines particularly deal with a safe application of wastewater with a focus on viral and microbiological risks. Some directions are given on how to assess fate and risks of contaminants, though in a generic manner. On the other hand, the current food safety legislation setting maximum limits (MLs) (e.g., 2023/915) only describes *known chemicals* in *known situations* and is therefore not equipped to handle new contaminants in unknown situations. Some reports have indicated no or little risk is to be expected in their field cases studied^36,49,50^, which is promising. However, this is far from a comprehensive understanding of risks in the diversity of crops, diversity of reclaimed water and diversity of agricultural production approaches. There is thus a need for a regulatory framework that supports a safe application of reclaimed water that also addresses chemical contaminants.

From the application of the five question approach, for a safe application of reclaimed water in food production, several knowledge gaps exist. These are related to the hazard (Q2), the fate (Q3), the risk (Q4) and the risk management (Q5).

With regard to the hazard identification (Q2), the knowledge gaps include the nature (identity) of contaminants in wastewater and the variation of contaminant levels (seasonal, batch-to-batch and between WWTPs). In this regard several studies reported on the identity and concentration levels of PFAS and other contaminants in (treated) wastewater^51–53^, but a substantial and systematic knowledge base is lacking. For many chemicals and physical hazards, only scarce information is currently available, particularly on reclaimed water intended for agricultural use.

With regards to the fate (Q3), knowledge gaps relate to the transmission of hazards among compartments, for example, to soil, crops and food or feed, taking into account the variation of these compartments (e.g., soil composition). For many hazards, current knowledge on the persistence and transmission of contaminants among compartments (e.g., mobility), is limited. Since it is not possible to study the fate of all chemicals in all circumstances, the ultimate aim is to derive models that allow to estimate the persistence and transfer to different compartments in different scenarios. When such a knowledge base is available, stakeholders can make relevant choices as towards what wastewater products can be used for what crops, and can rank the most urgent risks. This prioritization is urgently required to study the actual risk of hazards for humans, animals and the ecosystem upon exposure to the relevant concentrations of the hazards.

When considering PFAS, with regard to the risk (Q4) only limited information is available. Some studies demonstrate a negative effect on the human immune system, but these studies focused on PFOA and PFOS only. No or very limited information is available on all other PFAS which hampers the implementation of effective legislation. Furthermore, consequential risks, like a higher susceptibility for a microbial infection, are unknown.

In addition, technological developments are needed that support the removal of contaminants from wastewater products (Q5), or preferably, prevent contaminants from being disposed into wastewater. Another mitigation strategy is the monitoring of reclaimed water prior to application, preferably by a simple and on-site applicable methodology. To what degree these, and potentially other mitigation strategies, are cost effective remains to be determined.

### A way forward

Food systems reaching the planetary boundaries have led to the emergence of ambitious plans for reducing the impact on the planet. Circularity in food production can contribute substantially to achieving these goals. The transition to more sustainable food production is urgent. When it comes to circularity in food production, food safety is a prerequisite for societal acceptance of these processes. In the above described two cases, we highlight that there are several knowledge gaps, technical challenges and regulatory hurdles, or lacking regulations, that need to be resolved. Hurdles may counteract a timely transition to a more circular food production. Here we discuss possible solutions to this. A major challenge lies in the speed of transition versus careful and safe adaptation of processes. The discussion is particularly focused on possible solutions that enable a timely transition. This boils down to collecting relevant information, but also simplifying ways to characterize hazards, fate and risks.

As regards input (Q1), a better understanding is needed of the sources of co-products that can be valorized into food and feed production (case 1). Also information on composition, quality and safety of co-products and waste products is needed. This will facilitate a better direction towards a point of use in the food production chain. In that respect, a separated collection at the source will also enable a more specific use (directly utilizable, or after a biorefining step). Where current regulations prohibit use of such co-products (e.g., in the case of swill), the leverage of such regulation may stimulate the valorization of such products, once it has become clear that these streams can be used in a safe way. Monitoring of co-products for a wide range of known hazards is urgently needed. As regards case 2, a better understanding is needed of the wastewater products that may, after treatment, be used for irrigation or fertilization purposes.

As regards hazards (Q2), the current system for determining chemical hazards is mostly based on a substance-by-substance basis. The way forward here is in multiple directions. Chemical hazards should be regarded in the mixtures as they are relevant for exposure. Moreover, the rise of strong analytical measurement methodologies (e.g., high resolution mass spectrometry) demonstrate that exposure to a plethora of contaminants, degradation products and metabolites takes place simultaneously. Determination of all individual chemical, biological and physical hazards is unrealistic as it is resource and time-consuming. Prioritization of hazard analysis is required to move forward, as well as multi analytical techniques that can determine a range of contaminants in a sample at the time. The lack of pure chemical standards further complicates this situation. Therefore strategies like read-across, *in-silico* hazard determination and in-vitro hazard determination need to become accepted methodologies, also from a regulatory point of view^54–56^.

Concerning fate (Q3), there is a need to understand the circular food production systems as a whole. Lipophilic contaminants (e.g., dioxins) will follow the flow of the lipids in a food system, which allows for quick assessments where potential contaminants accumulate in a food system. It becomes more complex with chemical hazards which are intermediately lipophilic or water soluble and mobile, as these may end up in various compartments in the food chain and ecosystem. There is a clear need for a quick and easy assessment of the fate and persistence of contaminants (including uptake by crops, insects and animals), based on models that describe the relevant food production chains and compartments. Such models allow a quick assessment of the fate of the hazard in a temporal and spatial fashion. It is important to appreciate data on microbial, enzymatic, chemical and physical degradation as it may relieve possible hazards. In case more quantitative data are needed, such data should be gathered through dedicated experiments. The availability of powerful analytical techniques provides a wealth of data on the occurrence of known and unknown compounds (e.g., non-target screening & identification), and this can accelerate our understanding of the fate of contaminants. A generic model approach allows for an initial assessment of the fate, even when no identity or hazard of a substance has been assessed.

As regards risks (Q4), risk assessment has traditionally been based on a substance-by-substance basis. Given the large number of contaminants (e.g., PFASs), degradation products and metabolites, this calls for different approaches that integrate multiple contaminants at the same time, e.g., by looking at the adverse effect of groups of contaminants (mixture toxicity), by looking at certain physical-chemical properties of the compound(s) (quantitative structure-activity relationship = QSAR) or simply by taking representative marker contaminants that represent a complete class of compounds. New assessment methods (NAMs) may provide possibilities to assess larger numbers of contaminants at the same time. However, taking such approaches will lead to a higher level of uncertainty in the estimated risk, than the traditional substance-by-substance approach. In many cases, it is likely that there is sufficient margin to guarantee safety. In other cases, a need will emerge to reduce the uncertainty to get a more accurate estimation of the risk.

Risk management (Q5) is a very broad topic, that deals with development of food safety risk mitigation measures, monitoring, compliance testing etc. It is out of the scope of this paper to extensively discuss possible solutions. Briefly, regulations that have come into force in the past to protect consumers now hamper progress of the timely transition towards sustainable food production (case 1). On the other hand, a lack of a clear regulatory framework may hamper adaptation of new practices. For example, the lack of directions on how to deal with specifically chemical contaminants in reclaimed water (case 2) in relation to food safety when used for irrigation may prevent such water reuse. Furthermore, the transition to a circular food production system would benefit from cross-cutting approaches between different types of legislation including environmental, food safety, agricultural and health regulations. Such horizontal approaches should be based on quality protocols which set out ‘end of waste’ criteria for production as is now proposed in the revision of the Waste Framework Directive^57^.

Acknowledging the fact that regulations cannot be changed overnight, and that many stakeholders rely on current regulations, we pledge for re-evaluation of the food safety regulatory framework. Apart from regulatory aspects, there is also the aspect of acceptable risk: how much risk is acceptable for the stakeholders and society? There are promising methods and metrics to compare risks of various types, such as disability-adjusted life years (DALY)^58^. These approaches may help in the overall evaluation and comparison of different food safety scenarios. The discussion above focused on options to remove barriers for a speedy transition. Other viewpoints may also be relevant in the discussion. In the end it is part of the policy making process to balance these viewpoints.

### Concluding remarks

The transition towards a circular agro-food system is an important contributor to improve the sustainability of our food production system. How can we prevent the use of agrochemicals (e.g., plant protection products and veterinary drugs) in food production? And if these are required, how do we prevent accumulation and re-circulation of such contaminants in the ecosystem affecting aquatic and terrestrial biota health and soil health? Can biomass that was previously not applied for human food or animal feed be used as such without introducing a health hazard? And does the use of alternative sources or the reintroduction of co-products also reintroduce unwanted contaminants into the system? And can they accumulate? These are important safety-related aspects that must be considered in the current transition towards circular food production systems. These considerations help to prevent new safety related health risks, but they also contribute to the innovative and safe application of currently undervalued co-products, such as sewage sludge, swill and reclaimed water.

In current food production systems many food safety hazards are understood and controlled. It is anticipated that new hazards will appear or accumulate, leading to new -and less understood- food safety risks in the circular food production system. The current system of identification of hazards and the management thereof is not well-designed to support the fast transition to circular food production systems, that are expected to come rapidly over the next 5–15 years. Arriving at consensus if a certain contaminant is hazardous, and designing appropriate risk management measures may take 5–15 years of scientific, societal and political debate and assessment. Given the number of (potential) pathogens and chemicals of commerce, this is unacceptably long. We believe there should be a balance between a speedy transition and the safety of our food systems. Risk ‘acceptance’ (part of Q5) is, therefore, extremely relevant.

To assess, guide and accelerate the safety assessment of circular food production processes, a simple approach consisting of five questions is presented, complementing the generally accepted HACCP system. We pose that, even though the questions are straightforward, they are currently difficult to answer in many cases because we lack the knowledge to do so. Using this approach, we have identified knowledge gaps and regulatory hurdles that need to be resolved. In the transition to a circular economy, risk assessment and management should emphasize more on the exposure to unexpected (with regard to its nature and its origin) and mixtures of hazards, as hazards might circulate and accumulate in the food production system. Also we observe that more data on the occurrence and fate of hazards and the development of models are required to adequately perform risk assessment in a circular food production system. Last, new ways of valorisation of co-products are required in which a safe-by-design approach should be adopted.
